# Editorial: Post-translational modifications in human cancer: pharmacological insights and therapeutic opportunities

**DOI:** 10.3389/fphar.2025.1654659

**Published:** 2025-07-07

**Authors:** Yongzhi Chen, Angelina W. Fan, Lei Huang

**Affiliations:** ^1^ Division of Innate Immunity, Department of Medicine, University of Massachusetts Chan Medical School, Westborough, MA, United States; ^2^ Westborough High School, Westborough, MA, United States; ^3^ Department of Molecular, Cell, and Cancer Biology, University of Massachusetts Chan Medical School, Worcester, MA, United States

**Keywords:** post-translational modifications, PTMs, cancer, personalized medicine, PARP inhibitor, UPS inhibitor, SUMOylation inhibitors

Human cancer comprises a diverse and complex group of diseases defined by uncontrolled cell growth and proliferation. Among the key molecular mechanisms driving cancer progression, post-translational modifications (PTMs) of proteins play a pivotal role (Geffen et al. 2023; Li et al. 2023; Dutta and Jain, 2023). PTMs, chemical alterations that occur after protein synthesis, profoundly influence protein function, localization, stability, and activity within cells (Duan et al. 2024; Huang et al. 2022; Zhu et al. 2024). Targeting PTMs through pharmacological interventions represents a promising frontier in cancer therapy. Nevertheless, the successful development of such therapies demands a deep understanding of the specific PTMs involved and their roles in the molecular pathways of distinct cancer types. Moreover, personalized medicine is gaining prominence, with treatments increasingly tailored to the unique PTM signatures and genetic mutations of individual tumors. Unraveling the functional consequences of cancer-specific PTMs is essential for advancing precision-targeted therapeutic strategies. Our Research Topic explored PTMs in human cancer, spanning pharmacological insights to therapeutic opportunities, and featured both review articles and original research.Colorectal cancer remains a formidable global challenge (Biller and Schrag, 2021), and the study by Huang et al. brings fresh insight by spotlighting circRNA hsa_circ_0002238 as a potent oncogenic driver. Through rigorous *in vitro* and xenograft assays, the authors convincingly link elevated expression to accelerated tumor growth, epithelial-mesenchymal transition, and PI3K/AKT activation, while silencing reverses these hallmarks. Equally notable is the diagnostic promise and sex-specific enrichment in women, hinting at personalised screening strategies. Limitations include single-centre sampling and unanswered mechanistic questions on downstream effectors. Nevertheless, this work enriches our circRNA repertoire and paves the way for biomarker-guided therapies in colorectal cancer patients in clinical practice.

Jiang et al. conduct a comprehensive multi-omics analysis of osteosarcoma, integrating WGCNA-guided transcriptomics with robust experimental validation to enhance prognostic assessment. By spotlighting CHMP4C as the fulcrum of a 15-gene risk signature and elucidating its activation of p-GSK3β/β-catenin signaling, the authors bridge bioinformatics prediction and mechanistic proof. The integrated *in vitro* and xenograft assays convincingly link CHMP4C overexpression to enhanced proliferation, migration, and tumor growth, while knockdown reverses these traits. This work not only refines patient stratification but also nominates CHMP4C as a druggable node within the Wnt axis, inspiring therapeutic innovation against aggressive osteosarcoma in clinical practice and research.

Li and colleagues utilize a multi-omics strategy to reposition the food-derived dipeptide anserine as a vascular defender against deep-vein thrombosis. Integrating untargeted metabolomics, transcriptomics profiling, endothelial assays, and a vena-cava-ligation rat model, the study delineates anserine’s capacity to temper inflammation, restore nitric-oxide signaling, and extend coagulation times. Mechanistic experiments connect these benefits to MYB-centred PI3K/Akt modulation and CARNMT1-boosted anserine biosynthesis, forming a self-reinforcing metabolic loop. This work elevates anserine from nutraceutical curiosity to druggable lead, broadens the anticoagulant landscape beyond heparins and NOACs, and provides a blueprint for metabolite-guided therapy in thrombotic disease and paves paths for clinical translation.

Luo et al. interweave exercise biology and oncology in a data-rich inquiry that positions glycerophosphoinositol as a molecular switch synchronizing muscle adaptation with colon cancer restraint. Through cross-tissue GSVA, pan-cancer PPI, and methylome mining, they elevate FGA/NOTCH3 crosstalk as an exercise-responsive axis whose mis-wiring forecasts poor COAD prognosis. Wet-lab docking, qPCR, CCK-8, and apoptosis assays confirm that glycerophosphoinositol rewires NOTCH3 signaling, dampening growth while re-educating immune infiltration via PI3K/Akt modulation. By uniting omics, computation, and bench validation, the study reveals a metabolite/inflammation/ECM circuit ripe for biomarker deployment and suggests exercise-mimetic interventions that could personalize colon-cancer prevention and therapy in future precision oncology.

Integrating big-data bioinformatics with experimental validation, Shi et al. spotlight placental growth factor (PIGF) as a workout-responsive driver and drug target in head-and-neck squamous cell carcinoma. Their multi-omic pipeline, TCGA mining, single cell mapping, immune infiltrate deconvolution, and functional assays, links high PIGF to advanced stage, immune remodeling and biology, while silencing PIGF curbs proliferation and colony formation. Equally important, exercise-related gene signatures refine prognostication, hinting that lifestyle cues can inform therapy. This elegant synthesis propels PIGF toward precision oncology trials and underscores the translational dividends of coupling computational discovery with mechanistic validation. The study templates leveraging exercise biology to boldly tame tumor heterogeneity.

Feng et al. present a comprehensive genomic analysis of brain-metastatic lung adenocarcinoma, repositioning NLRP7 from an inflammatory sentinel to a metastasis suppressor and identifying the prostaglandin E antagonist AH-6809 as a promising inhibitor of epithelial-mesenchymal reprogramming. By integrating TCGA/GEO bioinformatics, pan-cancer methylome and ATAC-seq analytics with CCK-8, qPCR, colony formation, and immunofluorescence assays, the authors connect NLRP7 downregulation to EMT activation and show AH-6809 restores apoptosis, dampens SUMO1-mediated modification, and curbs metastatic traits. This elegant bench-to-database synergy refines prognostication, broadens the SUMOylation/EMT therapeutic axis, and offers a realistic route toward future clinical translation against brain-seeking LUAD metastasis, and inspires drug-development pipelines worldwide.

Guo et al. perform a comprehensive, multidimensional analysis of SUMOylation in prostate cancer, identifying NOP58 as a key driver of malignancy. Combining TCGA data, single-cell transcriptomics, spatial mapping, and experimental validation, they demonstrate that NOP58 overexpression is linked to poor survival, reprogrammed DNA repair and Myc signaling, and an immunosuppressive tumor microenvironment. Functionally, silencing NOP58 elevates ROS, triggers apoptosis, and reduces colony formation, while forced expression rescues these effects. Importantly, NOP58 overexpression sensitizes tumors to methotrexate, rapamycin, and sorafenib, positioning it as both a prognostic biomarker and a therapeutic target in SUMO-directed precision oncology for aggressive prostate cancer (Li et al., 2025). Furthermore, Min et al. repurpose the antibiotic paromomycin as an epigenetic therapy for glioblastoma. Bioinformatic analyses across cancers highlight SUMOylation machinery as a survival driver, and molecular docking identifies paromomycin as a potent HDAC1 antagonist. In glioblastoma U-251MG cells, paromomycin suppresses HDAC1-PIAS1 expression, reduces SUMO1 conjugation, inhibits colony formation and migration, and induces apoptosis via caspase-3. The drug also blocks IGF1R nuclear translocation, an effect reversible by trichostatin A, confirming HDAC1 dependence. This work repositions paromomycin as a potential glioblastoma therapeutic and underscores SUMO/HDAC crosstalk as a promising target for future drug development. Additionally, Zhang et al. integrate Mendelian randomization of over 1,400 metabolites with multi-omic cancer analyses to identify 57 metabolites causally linked to pulmonary hypertension (PH) and a 12-gene inflammatory/extracellular matrix signature shared across cancers. Functional assays reveal that omega-3 fatty acids mitigate ROS, suppress IL-6/IL-1β, inhibit SUMO1 nuclear transport, and reduce proliferation in diverse cancer cell lines. This study reframes PH as a metabolically modifiable, cancer-adjacent condition and positions metabolite-guided therapies and SUMOylation metrics as novel precision tools for cardio-oncology (Du et al., 2021; Long et al., 2020).

Li et al. provide the first quantitative synthesis showing that GSTP1 promoter hypermethylation serves as a potent molecular marker for hepatocellular carcinoma. By pooling 1,133 participants across ten studies, they show a six-fold enrichment of methylation in tumors and link the epigenetic silencing to advanced stage, recurrence, and inferior survival. Rigorous subgroup and sensitivity analyses bolster robustness, while moderate heterogeneity highlights avenues for methodological harmonization. The work positions liquid-biopsy assays for GSTP1 methylation as imminent companions to ultrasound and AFP, and argues that demethylating therapies could reactivate cellular detoxification defenses. This meta-analysis sets an actionable agenda for precision HCC screening and treatment.

Zhao et al. present a comprehensive overview of the m6A epitranscriptome in liver cancer, detailing how writers, erasers, and readers orchestrate hepatocarcinogenesis, metastasis, drug resistance, and immune remodeling. By integrating mechanistic studies across HCC, ICC, and hepatoblastoma, the authors clarify contradictory roles of METTL3, FTO, and YTHDF paralogues and spotlight context-specific vulnerabilities. The review’s therapeutic section is especially timely, evaluating FTO, METTL3, and ALKBH5 inhibitors, and proposing m6A-guided immunotherapy combinations. This authoritative synthesis will orient researchers, clinicians, and drug developers, and should catalyze the standardization of sequencing methodologies and accelerate the translation of epigenetic RNA editing into precision hepatology, worldwide patient benefit.

SIRT5 has evolved from a metabolic curiosity to a central regulator of onco-metabolism (Fabbrizi et al., 2023), and Ke et al. provide the most comprehensive roadmap to date. Their review traverses structural biology, NAD^+^ sensing, desuccinylation circuitry, and the Janus-faced roles of SIRT5 across hepatocarcinoma, lung, renal, and gastrointestinal malignancies. Particularly valuable is the integration of proteomic succinylomes with translational insights into quercetin, cyclic peptides, and MC3138, framing realistic drug-development trajectories. The authors also spotlight unanswered questions, contextual determinants of tumor promotion versus suppression, immune crosstalk, and combinatorial therapy that will chart the next wave of discovery. This authoritative synthesis is poised to advance precision metabolic oncology for both researchers and clinical innovators.
